# Near-Infrared Spectroscopy for the In Vivo Monitoring of Biodegradable Implants in Rats

**DOI:** 10.3390/s23042297

**Published:** 2023-02-18

**Authors:** Hafiz Wajahat Hassan, Eduarda Mota-Silva, Valeria Grasso, Leon Riehakainen, Jithin Jose, Luca Menichetti, Peyman Mirtaheri

**Affiliations:** 1 Faculty of Technology, Art and Design, Department of Mechanical, Electronic and Chemical Engineering, Oslo Metropolitan University, 0130 Oslo, Norway; 2 Institute of Clinical Physiology, National Research Council (IFC-CNR), 56124 Pisa, Italy; 3 Institute of Life Sciences, Sant’Anna School of Advanced Studies, 56127 Pisa, Italy; 4 FUJIFILM VisualSonics, 1114 AB Amsterdam, The Netherlands; 5 Faculty of Engineering, Institute for Materials Science, Christian-Albrecht University of Kiel, D-24143 Kiel, Germany

**Keywords:** biodegradable implants, in vivo monitoring, Mg alloys, near-infrared spectroscopy (NIRS), oxygen saturation (SO_2_), photoacoustic imaging (PAI), principal component analysis (PCA), tissue oxygen saturation (StO_2_)

## Abstract

Magnesium (Mg) alloys possess unique properties that make them ideal for use as biodegradable implants in clinical applications. However, reports on the in vivo assessment of these alloys are insufficient. Thus, monitoring the degradation of Mg and its alloys in vivo is challenging due to the dynamic process of implant degradation and tissue regeneration. Most current works focus on structural remodeling, but functional assessment is crucial in providing information about physiological changes in tissues, which can be used as an early indicator of healing. Here, we report continuous wave near-infrared spectroscopy (CW NIRS), a non-invasive technique that is potentially helpful in assessing the implant–tissue dynamic interface in a rodent model. The purpose of this study was to investigate the effects on hemoglobin changes and tissue oxygen saturation (StO_2_) after the implantation of Mg-alloy (WE43) and titanium (Ti) implants in rats’ femurs using a multiwavelength optical probe. Additionally, the effect of changes in the skin on these parameters was evaluated. Lastly, combining NIRS with photoacoustic (PA) imaging provides a more reliable assessment of tissue parameters, which is further correlated with principal component analysis.

## 1. Introduction

Biodegradable materials such as magnesium (Mg) and its alloys have increasingly been used in orthopedic surgery for bone fixation [1]. Although the cellular responses of surrounding tissue are important, their mechanism of action remains unclear [2]. Due to their osteogenic properties and biodegradability, Mg implants have been gaining significant attention among degradable materials as a superior alternative to bio-inert implants [3,4]. Titanium alloys are considered the gold standard for stabilizing fractured bones, also referred to as a bio-inert metallic implant [5]. Magnesium hydroxide temporarily inhibits osteoclastic activity and stimulates bone formation, but emphysema can occur if hydrogen gas production exceeds the tissue’s ability to absorb and transport the gas [6,7,8]. The formation and composition of the degradation layer alter physiological parameters such as the temperature, pH, ionic content, and protein content after implantation [9]. Currently, imaging techniques such as X-ray imaging, computed tomography (CT), synchrotron-radiation-based computer microtomography (SRCT), positron emission tomography (PET), PET-CT, magnetic resonance imaging (MRI), and ultrasound photoacoustic (USPA) can be used to assess Mg degradation and its effects in vitro/in vivo. However, these methods are costly and time-consuming, and not all of these techniques can be translated into a clinical context due to patient safety restraints [10,11,12,13]. Near-infrared spectroscopy (NIRS) technology enables the measurement of oxygen delivery, pH, and blood flow in tissues, which can be used to assess metabolic and respiratory health [14,15,16,17]. NIRS and partial least square regression (PLSR) can be applied to estimate the change in pH values in vivo and in vitro, as discussed in our previous studies [18,19].

NIRS is used as a rapid and cost-effective solution for in vivo monitoring [18,20]. NIRS allows for the non-invasive and continuous monitoring of tissue oxygenation [21]. Imaging techniques such as NIRS and USPA are commonly applied to soft tissues to access blood- and tissue-related properties [18]. As blood keeps cells in close contact with the external environment, measurements in the tissue bed can provide information about the interface between the implants and tissue. However, because the Hounsfield Unit (HU) of Mg alloys is similar to the bone structure, it is challenging to differentiate the Mg particles from bone tissue using microCT [22,23]. Therefore, NIRS can analyze Mg degradation’s effects, regardless of it having the same HU unit as bone, and in vitro studies have evaluated this degradation effect [19]. A device must be developed to be used in in vivo clinical procedures that can also be used in real-life scenarios to monitor the Mg alloy’s degradation effect. To the authors’ knowledge, no study has examined the tissues’ in vivo response to Mg reactions by using NIRS. As NIRS enables measuring oxyhemoglobin (HbO_2_) and deoxyhemoglobin (Hb) concentrations changes, information related to changes in tissue hemodynamics and oxygenation can be obtained [24]. Monitoring tissue oxygen saturation (StO_2_) can be used to measure peripheral hypoperfusion and tissue hypoxia [25,26]. Assessing StO_2_ in the implantation site is a relatively new approach to evaluating tissue healing [27,28]. The proposed study uses a novel optical probe with wavelengths from 650 nm to 1050 nm that probes into the specific region of interest at the implant tissue interface non-invasively. In one of our previous studies, we provided detailed information on the optical probe’s feasibility for optical phantoms [29]. An NIR optical probe can provide valuable information about the absorption reflectance properties of tissues. There are thousands of data points in a spectral dataset, many of which are noise, background, isosbestic, or dark regions or highly correlated information (i.e., broad absorption or emission bands) [30]. In order to visualize NIRS data, principle component analysis has been found to be a useful tool. PCA is commonly employed in NIRS studies to reduce data dimensionality, reduce noise impact, visualize data patterns, and identify trends that are not immediately evident, hence increasing accuracy and interpretation [30,31].

This study’s main objective was to verify the effectiveness of the NIRS technique when combined with Principal Component Analysis (PCA) for extracting information about tissue oxygenation and the correlation between different groups of spectral data when comparing Mg with titanium (Ti)-implanted animals and sham animals (animals without an implant, to which only bone defect was performed). In statistics, information is extracted from noisy data by converting it into a set of variables made up of uncorrelated linear combinations of the original variables (for example, principal components (PCs) containing most of the variability of the original variables within a dataset). Finally, photoacoustic imaging has validated the extracted tissue components [32].

## 2. Materials and Methods

**Figure 1:**
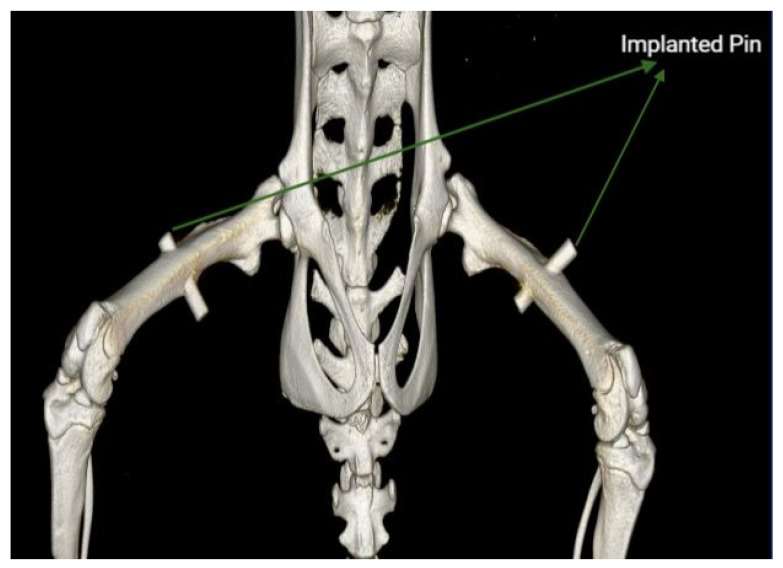
CT image showing the Mg implant positioning on day 7. Acquired with an IRIS PET/CT scanner (Inviscan Imaging System, Strasbourg, France), visualized in an OsiriX DICOM Viewer (Pixmeo, Switzerland).

In this pilot experiment, 15 12-week-old female Wistar rats were used. The animals were anesthetized with an intraperitoneal injection of 5 mg/kg Xylazine (Rompun Elanco, Italia, Florence, Italy) and 10 mg/kg Zoletil (Virbac, Opfikon, Gluttbrugg, Switzerland), while the depth of anesthesia was evaluated by the toe pinch response technique. Before surgery, the animal’s legs were shaved using a depilatory cream (Veet, Reckitt Benckiser Healthcare (Italia), Milano, Italy) and carefully cleaned to avoid skin burns from contact with the cream. An incision was made laterally on the leg, and the muscles were carefully teased away to expose the diaphyseal region of the femur. A drill with a 1.55 mm diameter was used to create a transcortical hole in the femur. A low drilling rotational speed was selected, and physiological saline (Fresenius Kabi, Verona, Italy) was dripped to minimize the frictional heat and thermal damage to the tissue. A pin implant with a length of 8 mm and a diameter of 1.6 mm made of either Mg-alloy (WE43) or Ti was implanted. The cylindrical implant was inserted by gentle tapping, resulting in a uniform press fit. After the pin placement, the wound site was cleaned with sterile cotton tips for the remaining bone debris, and the wound was closed with resorbable sutures (Johnson & Johnson Medical, Spa, Rome, Italy). The contralateral side was operated on similarly, using the same implant type. Figure 1 shows the implanted Mg pin in the CT image.

### 2.1. Experimental Workflow

**Figure 2:**
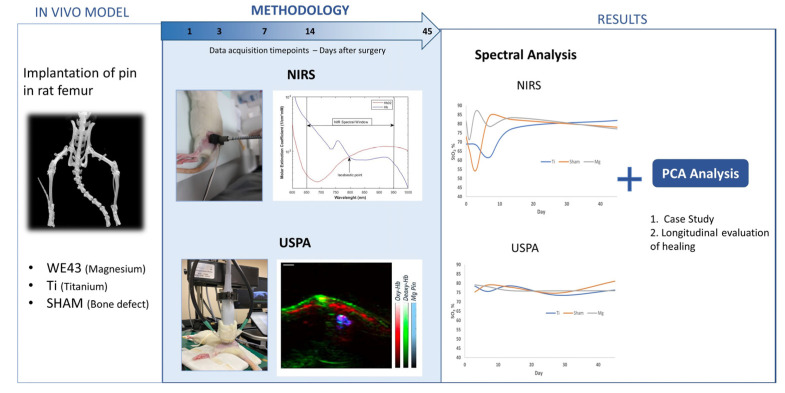
Illustration Summarizing the Workflow of the Study.

Our study involved implanting pins into rat femurs. To determine the oxygen saturation, we performed sequential NIRS and USPA acquisitions, followed by the application of MBLL on the NIRS data and of SPAX on the USPA data. We utilized PCA on the NIRS data to improve the presentation of the data for a short case study and a longitudinal evaluation of the bone-healing process. Our approach combines NIRS and PCA analysis and USPA validation to provide a comprehensive evaluation of the spectral measurements. Additionally, a flowchart was created to illustrate the steps involved in our study, as shown in Figure 2. The flowchart provides a clear visualization of the processes carried out, starting from the implantation of pins and ending with the analysis of the spectral measurements. In this study, oxygen saturation was measured using both USPA and CW-NIRS, resulting in the measurement of SO_2_ and StO_2_, respectively. The distinction between these two measurements arises from the differing principles and technologies utilized by each method.

### 2.2. NIRS Acquisition

**Figure 3:**
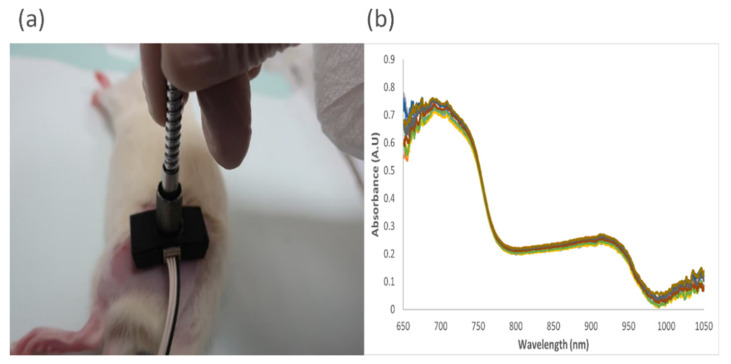
(**a**) The test bed for NIRS acquisition; (**b**) the raw absorption spectra for multiple measurements on a single point.

NIRS acquisitions were made prior to surgery, immediately after surgery, and on days 3, 7, 14, and 45. The animals were anesthetized with isoflurane (2.5% in pure oxygen) and placed laterally on a stereo-foam platform to help prevent rapid heat loss. When necessary, the regrowth fur was removed using the depilatory cream (Veet) prior to the acquisition. In this case, the acquisition was made 2–3 min after shaving to account for possible changes in the skin microcirculation due to friction while shaving and cleaning. The acquisitions were always made in the same room, with an average temperature of 25 °C, and on reduced luminosity conditions to reduce background noise. Figure 3a illustrates the NIR probe placement, and Figure 3b shows the obtained raw spectra with multiple acquisitions. The NIRS optical probe used for this experiment, which was developed at our lab at Oslo Metropolitan University, Oslo, Norway, has a source-detector distance of 8 mm. This specific value was chosen for a 3–3.5 mm penetration depth at the implant–tissue interface [29]. The optical light source of the probe operated in wavelengths between 650 and 1050 nm. To calculate the HbO_2_ and Hb, 740 nm and 840 nm wavelengths were chosen based on the prior differential path length factor (DPF) information from Geofrey D.V et al. [33]. The spectra data were collected from the Avantes Spectrometer (Avaspec-2048x14), which operates between 600 and 1100 nm. 

### 2.3. Modified Beer–Lambert Law (MBLL)

The modified Beer–Lambert law (MBLL) is the foundation of CW NIRS [34]. MBLL can be used to model what is known as CW wavelength-dependent diffuse reflectance [35].

(1) $$I\left(\lambda \right)=I_{0}\left(\lambda \right)e^{-\left(\mu_{a}\left(\lambda \right)\cdot DPF\left(\lambda \right)\cdot d+G\right)}$$

where I(λ) is the wavelength-dependent diffuse reflected light intensity, $I_{0}\left(\lambda \right)$ is the incident light, $\mu_{a}\left(\lambda \right)$ is the absorbance of the tissue, *d* is the source-detector distance, the differential pathlength factor is DPF(λ), and the constant *G* is a medium- and geometry-dependent constant. (1) can be written as:

(2) $$OD\left(\lambda \right)=-\ln\left(\frac{I_{0}\left(\lambda \right)}{I\left(\lambda \right)}\right)=\mu_{a}\left(\lambda \right)\cdot d\cdot DPF\left(\lambda \right)-G\;OD\left(\lambda \right)=-\ln\left(\frac{I_{0}\left(\lambda \right)}{I\left(\lambda \right)}\right)=\mu_{a}\left(\lambda \right)\cdot DPF\left(\lambda \right)\cdot d+G$$

where OD(λ) stands for optical density. When more than one chromophore contributes to the total absorption $\mu_{a}\left(\lambda \right),$ Equation (2) can be further rewritten as:

(3) $$OD\left(\lambda \right)=-\ln[\frac{I_{0}\left(\lambda \right)}{I\left(\lambda \right)}]=\sum_{i=1}^{n}[\varepsilon_{i}\left(\lambda \right)\;C_{i}]\cdot DPF\left(\lambda \right)\cdot d+G$$

where *n* is the number of chromophores, $\varepsilon_{i}\left(\lambda \right)\;$ is the extinction coefficient at the wavelength with the *i*th substance, and $C_{i}$ is the concentration of the given substance. Obtaining a total concentration estimate is impossible without knowledge of the geometry-dependent term. If the extinction coefficients $\varepsilon_{i}\left(\lambda \right)\;$are known for individual wavelengths, and measurements are treated differentially in time, it will be possible to cancel out *G* because it is usually assumed to be constant. Changes in the concentration are of primary interest in NIRS and are independent of *G*, specifically in CW-NIRS, unlike in the frequency domain or the time domain NIRS [36]. It is hypothesized that, at times t1 and t2, the extinction coefficient $\varepsilon_{i}$, DPF, D1, and G remain unchanged for measurements. The change in absorption is computed and removes factor *G*, resulting in a change in the concentration [36,37,38]. Equation (3) reduces to:

ΔOD(λ)=OD(t2,λ)−OD(t1,λ)

$$\Delta OD\left(\lambda \right)=\varepsilon \left(\lambda \right)\;C_{2}\cdot DPF\left(\lambda \right)\cdot d+G\left(\lambda \right)-\varepsilon \left(\lambda \right)\;C_{1}\cdot DPF\left(\lambda \right)\cdot d-G\left(\lambda \right)$$

ΔOD(λ)=ε(λ)·ΔC·DPF(λ)

(4) $$\Delta OD\left(\lambda \right)=\sum_{i=1}^{n}[\;\varepsilon_{i}\left(\lambda \right)\;C_{i}]\cdot DPF\left(\lambda \right)\cdot d$$

where Δ identifies the time-dependent changes. In order to approach the problem, at least two wavelengths are needed to calculate HbO_2_ and Hb, which are, in principle, the only absorbing compounds that show a time-dependent modulation. By rewriting Equation (4) in matrix format, we will have:

(5) $$\left[\begin{array}{c} \Delta OD\left(\lambda_{1}\right)\\ \Delta OD\left(\lambda_{2}\right) \end{array}\right]=d\cdot {\left[\begin{array}{cc} \varepsilon_{O_{2}Hb}\left(\lambda_{1}\right)DPF\left(\lambda_{1}\right) & \varepsilon_{Hb}\left(\lambda_{1}\right)DPF\left(\lambda_{1}\right)\\ \varepsilon_{O_{2}Hb}\left(\lambda_{2}\right)DPF\left(\lambda_{2}\right) & \varepsilon_{Hb}\left(\lambda_{2}\right)DPF\left(\lambda_{2}\right) \end{array}\right]}^{-1}\left(\begin{array}{c} \Delta O_{2}Hb\\ \Delta Hb \end{array}\right)$$

When measuring ΔOD from empirical measurements for the hemoglobin concentration, the above equation must be inverted. Please note that the a priori parameters of Equation (5) are the extinction coefficients of HbO_2_ and Hb at the two wavelengths *εi*(*λ*) and the DPFs at the two wavelengths. The geometrical inter-optode distance *d* and the changes in optical densities Δ*OD* are measured empirically.

$$\left(\begin{array}{c} \Delta HbO_{2}\\ \Delta Hb \end{array}\right)=\frac{1}{d}\frac{1}{\left[\begin{array}{cc} \varepsilon_{HbO_{2}}\left(\lambda_{1}\right)\varepsilon_{Hb}\left(\lambda_{2}\right) & \varepsilon_{Hb}\left(\lambda_{1}\right)\varepsilon_{HbO_{2}}\left(\lambda_{2}\right) \end{array}\right]DPF\left(\lambda_{1}\right)}$$

(6) $$\left[\begin{array}{cc} \varepsilon_{Hb}\left(\lambda_{2}\right) & -\frac{\varepsilon_{Hb}\left(\lambda_{1}\right)DPF\left(\lambda_{1}\right)}{DPF\left(\lambda_{2}\right)}\\ -\varepsilon_{HbO_{2}}\left(\lambda_{2}\right) & \frac{\varepsilon_{HbO_{2}}\left(\lambda_{1}\right)DPF\left(\lambda_{1}\right)}{DPF\left(\lambda_{2}\right)} \end{array}\right]\;\left[\begin{array}{c} \Delta OD\left(\lambda_{1}\right)\\ \Delta OD\left(\lambda_{2}\right) \end{array}\right]$$

A measurement of the tissue perfusion StO_2_ can be taken with NIRS by measuring changes in HbO_2_ and Hb concentrations, using the following predictors of the Hb and HbO_2_ relative concentrations:

(7) $$\;\;StO_{2}=\frac{HbO_{2}}{HbO_{2}+Hb}\times 100\%$$

Due to the assumption that the tissue scattering and pathlength remain constant throughout the measurement period, CW-NIRS does not provide absolute oxygen saturation values. The modified Beer–Lambert law is used instead to provide a semi-quantitative estimate.

### 2.4. Ultrasound and Photoacoustic Imaging

High-resolution Ultrasound (US) and Photoacoustic (PA) imaging (USPA) were acquired using the platform Vevo LAZR-X (FUJIFILM VisualSonics, Inc., Toronto, ON, Canada). The USPA imaging was performed sequentially after the NIRS measurement. This methodology was chosen to ensure that the results from the two techniques would be comparable and provide a comprehensive understanding of the samples. A linear US transducer array (MX 550) consisting of 256 elements at a nominal center frequency of 40 MHz and a bandwidth of 25–55 MHz is coupled with thin optical fibers and mounted on either side of the transducer. Homogenous light illumination is guaranteed by placing the sample to be imaged on the converging area of the two light beams. Spectral photoacoustic imaging (sPAI) was performed, within the wavelength range of 680–970 nm, with a 5 nm step. The imaging acquisition was made on days 3, 7, 28, and 45 after surgery. The rats were anesthetized with isoflurane (2–3% by volume with 0.8 L/min gas flow) and positioned in lateral recumbency, and the transducer was aligned perpendicularly to image the region of interest in the rats’ hindlimbs.

The automated detection of the HbO_2_ and Hb content was obtained from sPAI utilizing a newly developed superpixel data-driven unmixing (SPAX) framework, described in detail elsewhere [39]. The framework implements an SVD-based analysis to distinguish the relevant spectral information above the noise level automatically. SPAX is also extended to compensate for the spectral coloring artifact combining US image segmentation and spectral Monte Carlo (MC) light fluence simulations based on a predefined library of optical tissue properties. The advanced superpixel subsampling integrated within SPAX enables the detection of the least and most prominent components without a priori information.

Finally, the SO_2_ has been calculated from the normalized unmixed maps of HbO_2_ and Hb, obtained as the output of the SPAX analysis. Furthermore, the characteristic broad absorption spectrum of the metallic implant and its spatial distribution have also been obtained by the unsupervised SPAX approach.

### 2.5. Principal Component Analysis

Principal component analysis (PCA) was applied to NIRS data to extract practical information regarding the various factors affecting the Mg degradation. To perform exploratory analyses of NIRS data, PCA was applied on raw NIRS data. PCA is a mathematical procedure for resolving datasets into orthogonal components whose linear combinations approximate the original data by any desired degree of accuracy. The PCA method allows dominating patterns to be extracted from an NIRS data matrix. Two PCs were selected by examining the eigenvalues plot; outliers were detected and removed from the NIRS data after confirming their presence with spectral analysis. The dataset, including information about the implant types, rat group details, femur information (left and right), and timepoints (0, 3, 7, 14, and 45) was compiled. These data were preprocessed using normalization, one of the most commonly used preprocessing techniques. For subjects with damaged skin, a separate PCA was performed. In our study, PCA was performed using the open-source software R, in Rstudio.

## 3. Results

### 3.1. NIRS

**Figure 4:**
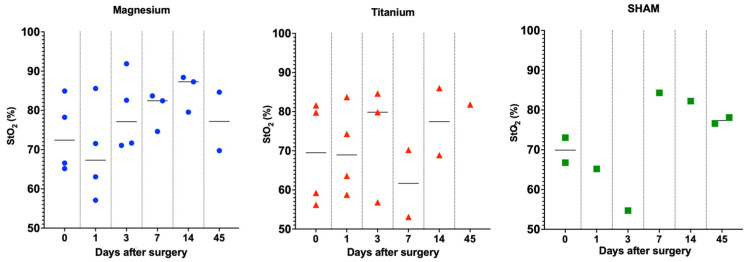
The tissue oxygen saturation (StO_2_%) values calculated for each animal for all timepoints (0, 1, 3, 7, 14, 45) are represented in the distribution plot.

StO_2_ was measured on the implanted site pre-operatively (day 0) and 1, 3, 7, 14, and 45 days postoperatively. On day 0, two measurements were taken pre-operatively and post-operatively. For the simplicity of the visualization of the data, pre-surgery is chosen as day 0, and post-surgery is written as day 1. The StO_2_ obtained from CW-NIRS for Mg-, Ti-, and sham-implanted rats is shown in Figure 4. Despite the reduced number of animals used for Ti-implanted and sham animals, it seems that Mg-implanted animals present a higher variability at the initial timepoints. The proposed study compared the spectroscopic characteristics of different implanted rats to assess StO_2_.

### 3.2. NIRS and USPA—Spectral Analysis

**Figure 5:**
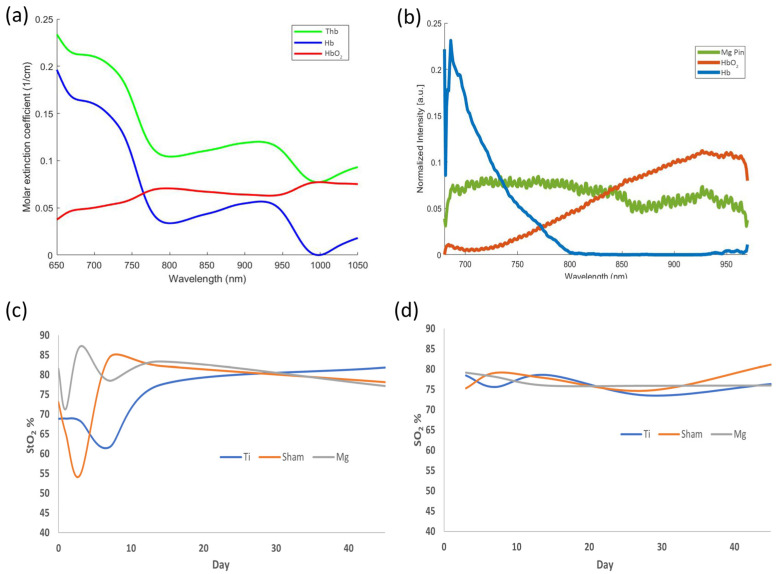
(**a**) The molar extinction coefficients of Hb, HbO_2_, and THb, as calculated from MBLL; (**b**) Unmixed spectra of the Hb, HbO_2_, and Mg implant pin after normalization; (**c**,**d**) Comparative analysis of oxygen saturation at the implant–tissue interface: (**c**) Near-infrared spectroscopy (NIRS); (**d**) Photoacoustic (PA) imaging.

The NIRS spectral region exhibited typical differences in the spectra of Hb and HbO_2_. Figure 4 shows how Hb and HbO_2_ have different wavelength dependences regarding extinction coefficients. The DPF values for the wavelength at 740 nm and 840 nm are 3.50 and 3.01, respectively, which are used to obtain the relative information about Hb and HbO_2_ based on the literature [33]. The wavelength dependency of blood absorption can be used to determine the blood StO_2_ level. The blue curve in Figure 5a represents the extinction coefficient of Hb, and the red and green curves represent the extinction coefficient of HbO_2_ and THb, respectively. Figure 5b shows the unmixing of the Hb, HbO_2_, and Mg pin from PAI. Figure 5c,d show a semi-quantitative analysis of the oxygen saturation calculated from spectral photoacoustic unmixing and NIRS. Here, we focused on NIRS evaluations, and USPA has been used to identify the anatomical structures and validate the molecular evaluations extracted from the entire region of interest. The graphs show a typical pattern of longitudinal variation in StO_2_ in the soft tissue around Mg and Ti implants. Similar trends have been retrieved from NIRS and USPA.

**Figure 6:**
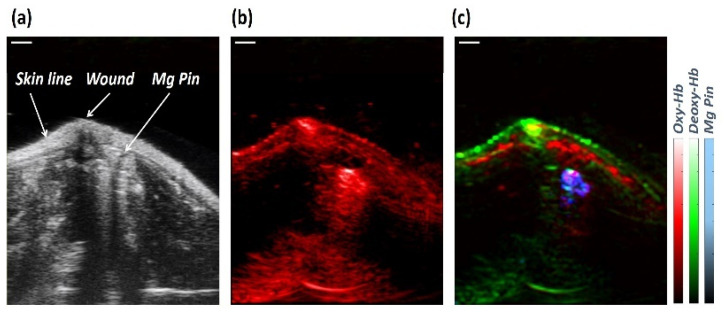
(**a**,**b**) The Ultrasound and Photoacoustic images of the Mg-implanted rat’s femur on day 3 after surgery in grey and red scales, respectively; (**c**) Overlapped unmixed distribution maps of the Oxy-, Deoxy-hemoglobin, and Mg pin in red, green, and blue scales, respectively, automatically obtained from sPAI. The scale bar size is 2 mm.

We used USPA imaging to study the possible presence of bubbles at the implant–tissue interface as a factor contributing to the higher variation in SO_2_ in the surrounding soft tissue of the Mg implant on day 3. Figure 6a,b show the ultrasound and photoacoustic images of the implanted rat femur at day 3, where the tip of the Mg pin is depicted in the short axes view. Figure 6c shows the spatial distribution of the unmixed components such as the HbO_2_, Hb, and Mg pin.

### 3.3. PCA Detects Significant Physiological Changes

#### 3.3.1. Case Study

**Figure 7:**
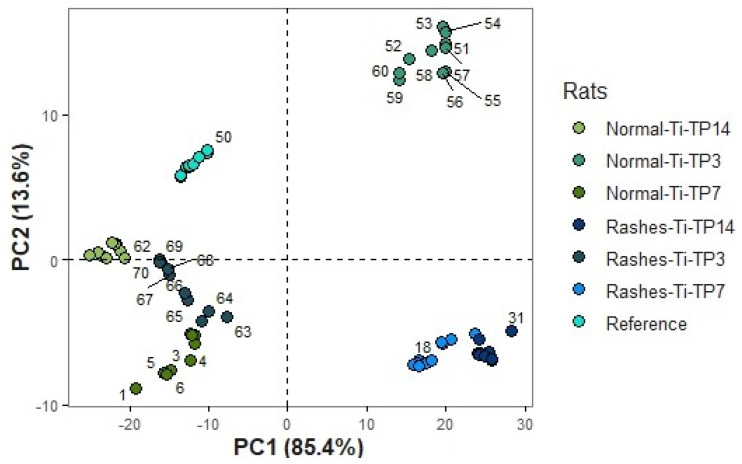
PCA score plot of normal vs. skin rash rats before surgery and on days 3, 7, and 14.

On day 3, one of the Ti-implanted rats developed a skin rash as an allergic reaction to the shaving cream that persisted until the end of the study. We found a measurable difference by analyzing the StO_2_ values and comparing a Ti-implanted animal with normal skin to a rat with skin rash. The data obtained at different timepoints were also visualized using PCA. In Figure 7, the PCA score plot shows the effect of damaged skin in terms of correlation at Timepoints 3, 7, and 14.

#### 3.3.2. Longitudinal Evaluation of Healing

**Figure 8:**
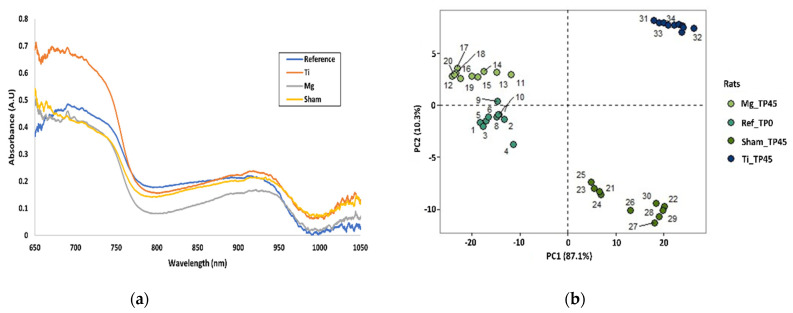
(**a**) Preliminary results in terms of absorption directly from the spectrometer, and (**b**) PCA score plot of all rats pre-operatively at Day 0 and post-operatively at Day 45; for reference, Mg-, Ti-, and sham-implanted rats are shown.

Furthermore, to assess the evolution of healing, we compared the measurements of the last timepoint, day 45, with the measurements performed before the surgery, day 0. The PCA score plot, represented in Figure 8, shows a significant spectral correlation at day 45 with day 0 on the Mg-implanted rats. Improbably, the Ti and Sham animals present a less significant correlation between the same timepoints. The same trend was observed in terms of the StO_2_ values of Mg-implanted rats; day 45 presented StO_2_ values similar to those of day 0. Although preliminary, these results suggest that there is a difference between Mg-implanted animals and Ti-implanted or sham animals.

## 4. Discussion

The modified Beer–Lambert approach is extensively used in the biomedical optics community due to its simplicity [35]. Researchers have used CW light to measure blood oxygenation and blood volume changes over time [24,37]. The present paper extends the modified Beer–Lambert approach to calculate the oxygen saturation of the tissues surrounding an implant. The NIR spectrometer Avaspec (Avantes, BV) can be used to measure the StO_2_ levels in the soft tissue located under a sensor probe to measure the oxygen content of the hemoglobin in the soft tissue. The spectra are captured in real time by radiating broadband NIR light, and only two selective wavelengths (740 nm and 850 nm) were used in MBLL to measure changes in HbO_2_ and Hb. Based on diffusion theory, it is possible to calculate the depth to which diffuse light will penetrate tissue as a function of the source-detector separation (d). Using a simple dependence, Weiss found that the photon depth before being detected is proportional to the square root of the source-detector separation (d) [40]. On average, the penetration depth of photons in the tissue is around 1/2 to 1/3 of the source-detector distance [41]. NIR light is estimated to reach a 3–3.5 mm depth, and the diffuse light is detected by the optical fiber inside the probe [29,42]. The same optical probe was recently used to validate the spectral absorption curves obtained from USPA [43]. As shown in Figure 5, we have used the molar extinction coefficient values to estimate the change in Hb and HbO_2_ using an optical probe developed at Oslo Metropolitan University, Oslo, Norway [29]. In order to investigate different wavelengths, we must have DPF values for each wavelength. Minor changes in DPF values could result in incorrect results [44]. It is assumed that these changes are minor, and, thus, a DPF value was chosen based on the literature that uses a DPF value based on the source-detector distance we selected.

NIRS studies have shown that oxygenation changes in the expected direction; however, absolute values can vary among studies and devices [45,46]. While CW-NIRS, such as the one used in this study, cannot estimate absolute concentrations, they are able to provide relative changes compared to a baseline, unlike FD-NIRS and TD-NIRS. The StO_2_ differences between some of the most commonly used commercial NIRS devices typically range from 10 to 15%, but they can be as high as 20% at low StO_2_ levels [46]. As a result of this variability across devices, it is challenging to establish thresholds for StO_2_ that can guide clinical management. Several equations were recently derived from converting values from one device to another to standardize oxygenation measurements across devices [45]. However, the StO_2_ values obtained through the NIRS probe, as reported in the proposed paper, are in line with the measurements obtained from USPA. One phenomenon associated with Mg implants degradation is the formation of gas bubbles around the implantation site, particularly at early timepoints after implantation, such as day 1 and day 3. This is challenging to measure with USPA, but NIRS presents a significant advantage in this sense. Therefore, NIRS can be used to investigate the bubble formation behavior of StO_2,_ particularly in the case of Mg alloy implantation. Upon implantation, magnesium and its alloys degrade in the body, releasing corrosion products such as H_2_ in the local environment [47]. For the in vivo study, maximum gas volumes were formed from earlier days until day 5 [48]. Rats implanted with Mg had the highest StO_2_ variation on days 1 and 3 relative to their baseline values on day 0 compared to rats implanted with Ti or sham implants.

Ultrasound (US) and photoacoustic (PA) imaging technology has been used to assess the SO_2_ changes in the vicinity of the implant. The combination of USPA with a recently developed unsupervised unmixing framework named superpixel photoacoustic unmixing (SPAX) has enabled the automated detection of the Mg-based implant, HbO_2_, and Hb as a prominent absorber [39]. Thus, this methodology was used as a comparison to benchmark the NIRS assessments in vivo. Although the sPAI unmixing provides results about the molecular tissue components’ spectra and their distribution maps, the SO_2_ evaluation is not an absolute measure. Thus, to obtain the absolute quantification in PA imaging, an improved measurement of the system response, optical tissue properties, and Grüneisen parameters are required. In the future, we aim to address the absolute quantification by implementing deep learning approaches based on non-explicit light fluence estimation, leading to high-fidelity SO_2_ estimations.

There have been a few studies that suggest that StO_2_ levels are significantly affected by the skin blood flow. NIRS measurements were shown to be affected by the skin blood flow by Buono et al. [49]. Our case study results showed that the animal with the skin rash presented a higher StO_2_ level associated with the abnormally high blood flow to the injured area. Furthermore, PCA was used in our study to demonstrate the potential for effectively differentiating between the spectral signatures collected from healthy and abnormal skin conditions. The results, although limited to one case, suggest the potential for PCA to be used in future studies to differentiate between healthy and diseased tissue. On a last note, our study uses albino rats, and the skin rash is easily detectable by visual observation; yet, NIRS could have more meaningful use in cases where skin pigmentation is higher and, thus, it is more difficult to detect changes visually.

In this study, we used PCA to detect differences between the timepoints (0, 1, 3, 7, 14, and 45). The PCA score plot for days 0 and 45 for all rats can be seen in Figure 7. Interestingly, on day 45, the rats implanted with Mg seemed closer to their pre-implanted state, day 0, when compared to the rats of the other groups. Moreover, the StO_2_ values of days 0 and 45 for the Mg-implanted animals were similar. This result seems to be in accordance with recent studies showing that Mg implants’ degradation promotes tissue regeneration and leads to a faster soft tissue homogeneity [47,50]. Although preliminary, our study shows that NIRS and PCA can detect these changes and are suitable tools for monitoring Mg implants degradation, particularly in an initial stage of soft tissue regeneration.

Our study presents a purely descriptive pilot study. However, to our knowledge, this study is the first longitudinal study using NIRS and PCA to assess, in vivo, the implant soft tissue–interface. With our NIR optical probe, we demonstrated that StO_2_ mainly changed postoperatively from day 0 to day 3, possibly due to bubble gas formation, a well-known side effect associated with the initial fast degradation of Mg implants. By using our approach, it is demonstrated that StO_2_ changes over time after implantation and that PCA can detect meaningful correlations with physiological reasoning. Further experiments with a larger group of animals should be conducted in order to significantly validate our findings. Furthermore, NIRS combined with multivariate techniques such as PLS and PCA and complemented with morphological information from USPA can provide a quick, objective, and reliable measure of the implant–tissue interface.

## 5. Conclusions

Applying the modified Beer–Lambert law (MBLL) to particular measurement conditions related to the effect of Mg degradation within tissues can enhance the reliability of data on the target tissue’s physiological state and biochemical content. NIRS is a non-destructive, non-invasive technique used to monitor tissue oxygenation in various clinical settings. This study shows that it may be a valuable method for measuring tissue oxygen levels and monitoring the tissue healing associated with Mg implant degradation. We use NIR spectroscopy techniques along with PCA analysis to assess the tissues surrounding the implant–tissue interface over time and detect significant changes. By combining NIRS and USPA, many other tissue parameters can be measured non-destructively and non-invasively. Furthermore, NIRS combined with multivariate techniques such as PLS and PCA and complemented with morphological information from USPA can provide a quick, objective, and reliable measure of the implant–tissue interface.

## Data Availability

Not applicable.
